# The associations of Positive and Negative Valence Systems, Cognitive Systems and Social Processes on disease severity in anxiety and depressive disorders

**DOI:** 10.3389/fpsyt.2023.1161097

**Published:** 2023-06-16

**Authors:** Bernd R. Förstner, Sarah Jane Böttger, Alexander Moldavski, Malek Bajbouj, Andrea Pfennig, André Manook, Marcus Ising, Andre Pittig, Ingmar Heinig, Andreas Heinz, Klaus Mathiak, Thomas G. Schulze, Frank Schneider, Inge Kamp-Becker, Andreas Meyer-Lindenberg, Frank Padberg, Tobias Banaschewski, Michael Bauer, Rainer Rupprecht, Hans-Ulrich Wittchen, Michael A. Rapp, Mira Tschorn

**Affiliations:** ^1^Social and Preventive Medicine, Department of Sports and Health Sciences, University of Potsdam, Potsdam, Germany; ^2^Department of Psychiatry and Psychotherapy, Central Institute of Mental Health, Medical Faculty Mannheim, Mannheim, Germany; ^3^Charité–Universitätsmedizin Berlin, Department of Psychiatry, Humboldt-Universität zu Berlin and Berlin Institute of Health, Berlin, Germany; ^4^Department of Psychiatry and Psychotherapy, University Hospital Carl Gustav Carus, Technische Universität Dresden, Dresden, Germany; ^5^Department of Psychiatry and Psychotherapy, University of Regensburg, Regensburg, Germany; ^6^Max Planck Institute of Psychiatry, Munich, Germany; ^7^Institute of Clinical Psychology and Psychotherapy, Technical University Dresden, Dresden, Germany; ^8^Translational Psychotherapy, Institute of Psychology, University of Goettingen, Goettingen, Germany; ^9^Department of Psychiatry and Psychotherapy CCM, Charité–Universitätsmedizin Berlin, Humboldt-Universität zu Berlin and Berlin Institute of Health, Berlin, Germany; ^10^Department of Psychiatry, Psychotherapy and Psychosomatics, Faculty of Medicine, RWTH Aachen University, Aachen, Germany; ^11^JARA-Brain, Research Center Jülich, Jülich, Germany; ^12^Institute of Psychiatric Phenomics and Genomics (IPPG), University Hospital, LMU Munich, Munich, Germany; ^13^Department of Psychiatry and Behavioral Sciences, Norton College of Medicine, SUNY Upstate Medical University, Syracuse, NY, United States; ^14^Department of Psychiatry and Behavioral Sciences, The Johns Hopkins University, Baltimore, MD, United States; ^15^University Hospital Düsseldorf, Medical School, Heinrich Heine University Düsseldorf, Düsseldorf, Germany; ^16^Department of Child and Adolescent Psychiatry, Psychosomatics and Psychotherapy, Philipps University Marburg, Marburg, Germany; ^17^Department of Psychiatry and Psychotherapy, University Hospital, LMU Munich, Munich, Germany; ^18^Department of Child and Adolescent Psychiatry and Psychotherapy, Central Institute of Mental Health, Medical Faculty Mannheim, Mannheim, Germany

**Keywords:** Research Domain Criteria, depression, anxiety disoders, disease severity, transdiagnostic, RDoC

## Abstract

**Background:**

Anxiety and depressive disorders share common features of mood dysfunctions. This has stimulated interest in transdiagnostic dimensional research as proposed by the Research Domain Criteria (RDoC) approach by the National Institute of Mental Health (NIMH) aiming to improve the understanding of underlying disease mechanisms. The purpose of this study was to investigate the processing of RDoC domains in relation to disease severity in order to identify latent disorder-specific as well as transdiagnostic indicators of disease severity in patients with anxiety and depressive disorders.

**Methods:**

Within the German research network for mental disorders, 895 participants (*n* = 476 female, *n* = 602 anxiety disorder, *n* = 257 depressive disorder) were recruited for the Phenotypic, Diagnostic and Clinical Domain Assessment Network Germany (PD-CAN) and included in this cross-sectional study. We performed incremental regression models to investigate the association of four RDoC domains on disease severity in patients with affective disorders: Positive (PVS) and Negative Valance System (NVS), Cognitive Systems (CS) and Social Processes (SP).

**Results:**

The results confirmed a transdiagnostic relationship for all four domains, as we found significant main effects on disease severity within domain-specific models (PVS: *β* = −0.35; NVS: *β* = 0.39; CS: *β* = −0.12; SP: *β* = −0.32). We also found three significant interaction effects with main diagnosis showing a disease-specific association.

**Limitations:**

The cross-sectional study design prevents causal conclusions. Further limitations include possible outliers and heteroskedasticity in all regression models which we appropriately controlled for.

**Conclusion:**

Our key results show that symptom burden in anxiety and depressive disorders is associated with latent RDoC indicators in transdiagnostic and disease-specific ways.

## 1. Introduction

Major depressive (MDD), as well as anxiety disorders (AD) may be characterized by altered emotional processes expressed upwards from neural circuitry to clinically relevant variations of symptomatology. On symptom level, MDD and AD share common features of aberrations of mood and emotions. On the one hand, high negative affect is present in both types of disorders, with depressed mood/anhedonia as well as anxious mood associated with both MDD and AD. On the other hand, anxious hyperarousal and persistent fear, anxiety or avoidance of perceived threats are considered general characteristics of AD, whereas low positive affect is relatively specific to MDD and only to certain distress-related types of AD, such as social anxiety disorder (SAD) or generalized anxiety disorder (GAD) (1–3). Furthermore, symptoms of anhedonia, meaning the loss of pleasure or interest in previously rewarding activities, are strongly tied to MDD. There is also an association of cognitive dysfunction for both disorders, while this association is more heterogenous for AD due to its broad disease spectrum (4). Existing literature also shows heterogeneous associations with respect to social processes. For example, the construct of affiliation and attachment has been associated with MDD and SAD, whereas the construct of understanding of self and others has been associated with GAD (5).

Common features in symptomatology and common neurobiological mechanisms in depressive and anxiety disorders can be considered partly responsible for limitations in diagnostic specificity, which is necessary to develop precise treatments (precision medicine) that can improve the stagnant treatment of mental illness.

The Research Domain Criteria (RDoC) approach promoted by the National Institute of Mental Health (NIMH) aims to address these issues and guide research toward a better understanding of mental disorders and their underlying psychological, neural and biological mechanisms, ultimately leading to improved treatments. The RDoC approach views mental disorders as syndromes at multiple levels, also connected to disrupted or dysfunctional brain circuitry (6, 7). To gain a better understanding of the links between disease-specific symptomatology and the underlying neural mechanisms of emotional (dys) function, the latent RDoC domains Positive (PVS) and Negative Valance System (NVS), Cognitive Systems (CS) and Social Processes (SP) were established and proved to be valid research constructs (8–12).

The PVS domain includes mechanisms involved in responses to attractive stimuli, such as responding to reward, as well as learning and valuation of rewards as parts of the reward system. In contrast, the NVS domain comprises responses to aversive stimuli of acute, potential, and sustained threat, loss, or aggression due to frustration. The CS domain comprises of circuits generating attentional processes, perception, memory functioning, language processing and cognitive control. The SP domain contains concepts of affiliation and attachment, social communication, as well as perception and understanding of self and others (13). In our previous research, we identified four distinct domains (PVS, NVS, CS, SP) in a latent structure of four overlapping factors (12).

There is limited research on PVS functioning within the spectrum of anxiety disorders, with most studies focusing on patients with specific anxiety disorders such as SAD and GAD, e.g., (14–16). These studies suggest that individuals with SAD and GAD tend to have reduced positive experiences and use experiential avoidance as a coping mechanism. However, PVS-related processing has been extensively studied in mood disorders. Symptoms of anhedonia in MDD have been associated with blunted reactivity to positively valanced and rewarding stimuli, e.g., (17–20), as well as hypoactivation of brain circuits linked to those stimuli, e.g., (21–23). In summary, existing literature on both types of disorders highlights disease-specific and therefore distinct profiles of reward processing.

Across units of behavioral, physiological, and neuronal data, there is ample evidence of similar NVS-related processing in MDD and AD: AD has been associated with a negativity bias toward negatively valanced stimuli, e.g., (24–26), and altered activity in brain structures associated with responses to threat-related stimuli, e.g., (27, 28); analogously, MDD has also been associated to a bias toward negatively valanced stimuli, e.g., (21, 29, 30), and threat-related negative stimuli, e.g., (31).

The occurrence of cognitive deficits regarding attention, memory and executive functioning in AD and MDD is well established (4, 32). However, the differentiation of disease-specific neural circuitry is challenging due to the lack of transdiagnostic and multimodal research (33) and because heterogeneous evidence exists for disorder-specific circuit alterations (3, 34).

While subconstructs of SP like attachment could be associated with social anxiety for example (35), the general impact of SP on AD is unclear due to the broad construct spectrum of SP in combination to the heterogenous disease patterns. Yet, the role of SP in specific types of AD such as SAD, has been more extensively investigated. This is because its symptomatology is closely linked to these processes, such as dysfunction in automatic association to social cues (36). As for MDD, impairment of social functioning is an evident sign and part of the structure of the disease. Kupferberg and colleagues (37) summarized that all SP subconstructs are impaired in patients with depression, hyper-sensitivity to social rejection, competition avoidance and increased altruistic punishment regarding the affiliation and attachment subconstruct, impaired emotion recognition, diminished cooperativeness regarding social communication and lastly reduced empathy or theory-of-mind deficits regarding social perception.

In recent years, there has been growing interest in transdiagnostic research approaches [e.g., (38)]. Recent studies aimed to provide evidence for transdiagnostic and disorder-specific psychopathological endophenotypes of NVS-related abnormal threat processing in AD and MDD (33, 39), an attentional bias to negative stimuli in AD and MDD (40–42), as well as PVS-related impaired reward functioning in MDD that is phenomenologically characterized by anhedonia (33, 41, 43). Regarding PVS on the domain level, low levels of positive emotions at a global level have been identified as risk factors for MDD, SAD, and GAD (44, 45).

Using the RDoC approach to investigate transdiagnostic markers of disease severity could help clarify whether mechanisms associated with PVS, NVS, CS and SP contribute to disease severity. Consequently, investigating how individual differences across RDoC domains (PVS, NVS, SP, CS) explain variance in disease severity, could enhance our understanding of possible mechanisms contributing to disease severity. Dimensional assessment of these four domains could help modify classical diagnostic categories and furthermore, it could inform the development of individualized precision treatment for psychiatric disorders (7, 42).

The main aim of this study was to investigate PVS, NVS, CS and SP processing in relation to disease severity implemented into a transdiagnostic and dimensional approach. We thereby aim to improve the understanding of underlying mechanisms of the AD and MDD disease spectrum and shed light on disease-specific as well as transdiagnostic indicators of disease severity. To the best of our knowledge, to date, no study has yet focused on testing RDoC domains as indicators of disease severity in psychiatric disorders. Therefore, our research focuses on both the relationship between the four RDoC domains and transdiagnostic disease severity, as well as the domains diagnosis-specific effects. We hypothesized that PVS, CS, SP would be negatively associated with disease severity, while NVS would be positively related with disease severity. Second, we predicted that PVS, CS and SP would have a disease-specific relationship with disease severity, while all four domains were expected to also show a general transdiagnostic relationship with disease severity.

## 2. Methods

### 2.1. Participants

This investigation is an observational cross-sectional study assessing four core domains of the RDoC matrix (PVS, NVS, CS, and SP) within the German research network for mental disorders [Forschungszentrum zu psychischen Erkrankungen; FZPE] (46) as outlined by Förstner et al. (12). Study centers throughout the FZPE network recruited participants for clinical and observational studies. A minimal RDoC test battery covering behavioral and self-report units of analysis was incorporated into the existing assessment process at baseline to evaluate the aforementioned RDoC domains. The process of data collection and processing has been previously described in further detail (12). A subsample of patients with a primary diagnosis of major depression (MDD; ICD-10: F32, F33, F34.1) or an anxiety disorder (AD; ICD-10: F40, F41) (*N* = 859) was selected for analysis (see Table 1 and Supplementary Tables S1, S2 for sample characteristics). Diagnoses were determined by expert clinicians in accordance with the 10th Revision of the International Classification of Diseases [ICD-10; World Health Organization (WHO) (47),] and/or the Fourth Edition of the Diagnostic and Statistical Manual of Mental Disorder [DSM-IV; (48)]. On average, patients with AD were younger than patients with MDD, had a higher number of comorbidities, and were more likely to be married. Regarding comorbidities 54.53% (*n* = 289) of patients with AD had comorbid MDD [diagnostic data from CIDI interview only (49)] and 23.08% (*n* = 12) of patients with MDD had comorbid AD. A greater proportion of patients with MDD were receiving psychotropic medication. There were no further significant differences between AD and MDD patients with respect to gender and sociodemographic variables, including education.

**Table 1:** Sample characteristics.

Variable	Sample			*p*
	Overall (*N* = 859)	AD (*n* = 602)	MDD (*n* = 257)	
Gender, *N* (%)				0.812
Female	476 (55.4)	332 (55.1)	144 (56.0)	
Age, y				
*M* ± SD	35.05 ± 12.83	32.94 ± 11.21	40.02 ± 14.89	<0.001
Range	15–78	15–68	18–78	
Marital status, *N* (%)				<0.001
Single	352 (40.0)	235 (39.0)	117 (45.5)	
Married/partnership	379 (44.1)	318 (52.8)	61 (23.7)	
Separated	20 (2.3)	8 (1.3)	12 (4.7)	
Divorced	60 (7.0)	39 (6.5)	21 (8.2)	
Widowed	4 (0.5)	2 (0.3)	2 (0.8)	
Missing	44 (5.1)	–	44 (17.1)	
Graduation, *N* (%)				0.939
Still in school	9 (1.1)	3 (0.5)	6 (2.3)	
CSE	75 (8.7)	54 (9.0)	21 (12.1)	
GSCE	220 (25.6)	171 (28.4)	49 (19.1)	
Polytechnic degree	6 (0.7)	3 (0.5)	3 (1.2)	
Technical-diploma	87 (10.1)	72 (12.1)	15 (5.8)	
University-entrance diploma	429 (53.4)	286 (47.5)	143 (55.6)	
Other	2 (0.2)	2 (0.3)	–	
School dropout	18 (2.1)	10 (1.7)	8 (3.1)	
Missings	13 (1.5)	–	13 (0.8)	
Occupation, *N* (%)				0.350
Employed	533 (62.1)	416 (69.1)	117 (45.5)	
Unemployed	295 (34.3)	186 (30.9)	109 (42.4)	
Missings	31 (3.6)		31 (12.1)	
Clinical characteristics				
Comorbidity, *N* (%)	582 (67.8)	530 (88.0)^a^	52 (20.2)	<0.001
Psychotropics, *N* (%)	521 (60.7)	296 (49.2)	225 (87.5)	<0.001

To compare patient groups appropriate analyzes were performed. *M*, Mean; SD, Standard deviation; y, years. AD, Anxiety disorders; MDD, Major depressive disorders. CSE, Certificate of Secondary Education [Hauptschulabschluss]; Polytechnic degree, [Abschluss der allgemeinbildenden Polytechnischen Oberschule der ehemaligen DDR]; GCSE, General Certificate of Secondary Education [Realschulabschluss]; Technical-diploma, [Fachabitur, Fachhochschulreife, Fachgebundene Hochschulreife]; University-entrance diploma, [Abitur, Allgemeine Hochschulreife]. ^a^Only indication of comorbid AD.

### 2.2. Self-report and behavioral RDoC operationalization

The four RDoC domains PVS, NVS, CS and SP were represented as individual patient factor scores from the four-factor CFA that had been conducted previously. Standardized factor scores were estimated using a linear regression method as reported by Förstner et al. (12). For ease of interpretation, factor scores were recoded positively, so that higher scores indicate higher expressions of the assessed domain. Therefore, higher scores in PVS indicate greater hedonic affect, and higher NVS scores indicate higher levels of anxious affect and somatization. Higher CS scores indicate better executive control, attention and working memory and higher SP scores indicate increased social skills, less interpersonal hostility and sensitivity, less paranoid ideas and less social anhedonia. For further details regarding the factor score composition, see Supplementary Table S3 (12). Table 2 provides sample details on the domain scores and the outcome variable disease severity, which is described below.

**Table 2:** Characteristics of domain-factor scores and disease severity.

	*M*	SD	Min	Max	Mdn	IQR	AD (*M*, SD)	MDD (*M*, SD)	*p*
PVS	−0.29	0.91	−3.31	1.29	−0.14	1.25	−0.23, 0.86	−0.41, 1.01	<0.01
NVS	0.37	0.90	−1.16	3.61	0.21	1.19	0.46, 0.83	0.15, 1.00	<0.001
CS	−0.09	0.87	−5.46	2.75	0.05	0.91	−0.07. 0.73	−0.12, 1.13	–
SP	−0.30	0.94	−3.36	1.16	−0.11	1.31	−0.28, 0.91	−0.33, 1.01	–
DS z-score	−0.35	1.16	−3.81	3.34	−0.37	1.14	−0.03, 0.78	−1.12, 1.49	<0.001

Total *N* = 859; PVS, Positive Valance System factor score; NVS, Negative Valence Systems factor score; CS, Cognitive Systems factor score; SP, Social Processes factor score; DS, Disease severity; *M*, Mean; SD, Standard deviation; Min, Minimum Score; Max, Maximum score; Mdn, Median; IQR, Interquartile range.

### 2.3. Disease severity assessment

Disease severity was assessed using disease-specific symptom-based self-report scales (4.7%), observer ratings (85.4%) or expert-based global rating scales (9.9%). To serve as a transdiagnostic outcome variable, all disease-specific severity values were z-standardized considering normative data from adult clinical samples. These samples had to meet the following criteria (1): provide a baseline distribution for the specific disease severity score, (2) contain as closely as possible represent the reference population (e.g., patients with MDD), and (3) contain a minimum of 500 participants and be representative if possible. Supplementary Table S4 provides detailed information on the normative data that was used for z-transformation.

### 2.4. Statistical methods

Several simple and multiple Linear Models (LM) were used (models 0–6) in this analysis with step-by-step insertion of type of diagnosis (dichotomous variable) as fixed-effect, followed by PVS, NVS, CS and SP factor scores as continuous independent covariates, and followed by PVS by diagnosis, NVS by diagnosis, CS by diagnosis and SP by diagnosis (factor-covariate) interactions, and disease severity z-score as the dependent outcome. To address the overlapping structure identified in the previous CFA (12), we controlled for multicollinearity in the models m1 and m2. Since multicollinearity was present in both models, we decided to perform further analyzes on domain-specific models (m3-6) by including diagnosis as fixed effect, the specific domain as an independent covariate, and their respective interaction (f.e., m3: disease severity ~ diagnosis + PVS + PVS by diagnosis).

The Shapiro-Wilks test, which was used to check for normal distribution of variables, indicated that all 5 variables were significantly different from a normal distribution (*p* < 0.001). Since our sample size largely exceeded the central limit theorem cut-off (*N* > 30), these deviations can be considered acceptable. To identify possible outliers, grouped boxplots were used for independent variables. Furthermore, Cook’s Distance (50) was used to identify influential data points in the analyzed regression models [Di > 0.85 (51)]. No data were removed as no data point exceeded the cut-off in any model. Levene’s test showed that equal variances between groups (AD vs. MDD) could be assumed for SP but not for disease severity (*p* < 0.001), PVS (*p* < 0.05), NVS (*p* < 0.01) and CS (*p* < 0.001). Breush Pagan tests were used to check for homoscedasticity. When heteroscedasticity was present, a suitable heteroskedasticity-consistent (HC) covariance estimation method (f.e., 52) was used in addition. All analyzes were performed using R version 4.2.2 with RStudio 2022.07.2 Build 576.

## 3. Results

**Figure 1:**
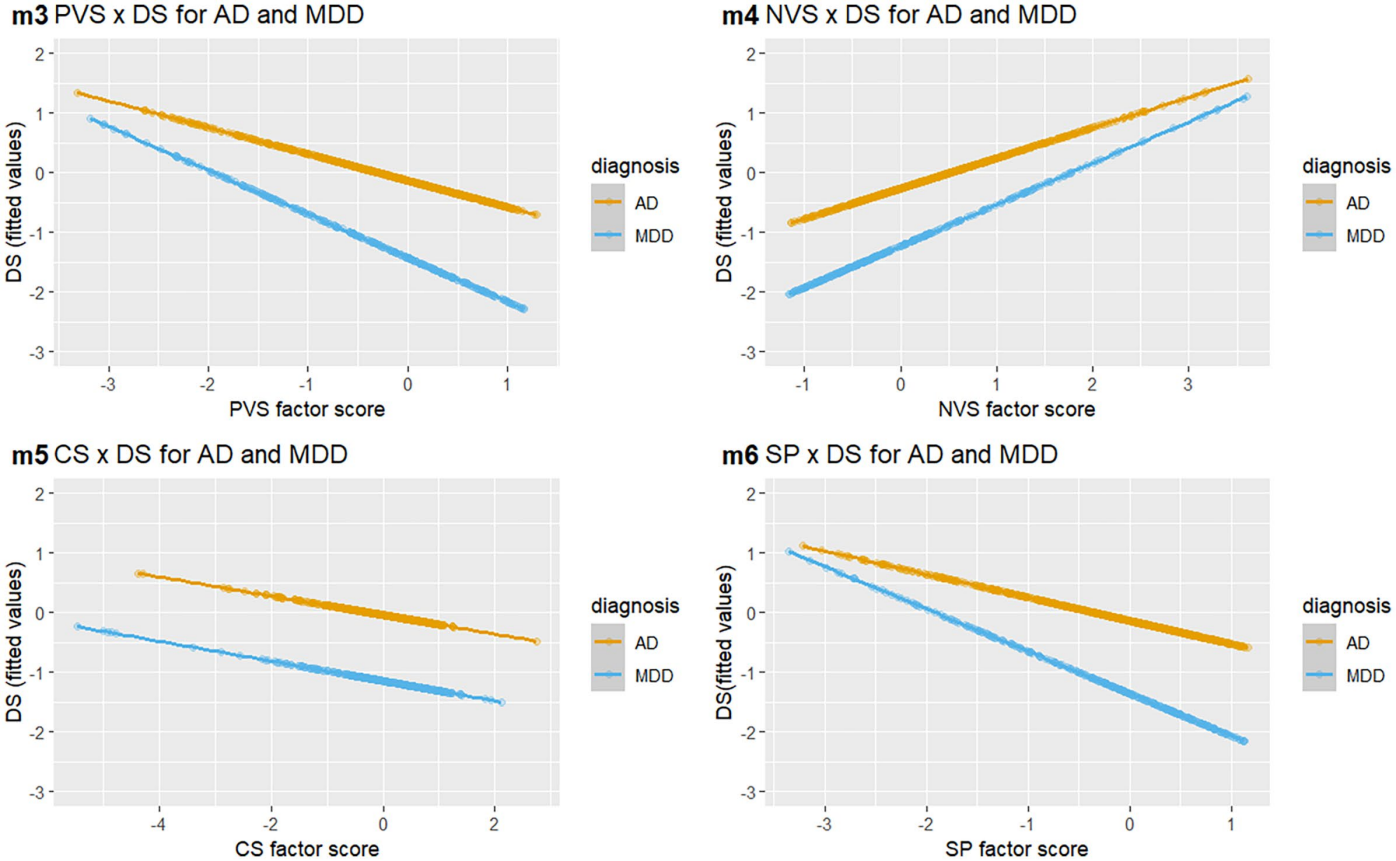
Relationship of RDoC domains with disease severity in AD and MDD. Grouped scatter graph of domain associations (PVS, NVS, CS, and SP) with fitted DS scores. Each dot corresponds to an individual score on both variables, the color represents the patient groups (orange: AD; blue: MDD). RDoC, Research Domain Criteria; AD, Anxiety disorders; MDD, Major depressive disorders; DS, disease severity; PVS, Positive Valence Systems; NVS, Negative Valence Systems; CS, Cognitive Systems; SP, Social Processes.

We performed incremental linear regression models (LM) in four steps starting with a simple LM containing only main diagnosis and disease severity [m0: *R*^2^ = 0.19; *F*(1,857) = 198.70, *p* < 0.001]. Main diagnosis significantly predicted disease severity (*β* = −0.43; *p* < 0.001) with higher scores of disease severity in the AD group compared to the MDD group. In the next step (m1), we added all four RDoC domain factor scores as independent covariates to the m0 equation [*R*^2^ = 0.41; *F*(5,853) = 116.80, *p* < 0.001]. M1 revealed significant main effects for main diagnosis (*β* = −0.42; *p* < 0.001), PVS (*β* = −0.37; *p* < 0.001), NVS (*β* = 0.30; *p* < 0.001) and SP (*β* = 0.18; *p* < 0.05). The previous effect of diagnosis remained significant and additionally PVS was negatively associated to disease severity, while NVS and SP were positively associated with disease severity. We found no significant main effect of CS on disease severity. To control for multicollinearity, we calculated variance inflation factors (VIF) for m1. PVS (VIF = 10.04) and SP (VIF = 10.74) exceeded the cut-off (VIF > 10) indicating a high correlation of those predictors. Compared to m0, m1 showed a significantly better fit [*F*(4,853) = 78.38, *p* < 0.001] and larger *R*^2^. In a third step (m2), we added the four interactions of the domains with main diagnosis to m1 [*R*^2^ = 0.42; *F*(9,849) = 67.01, *p* < 0.001] to assess additional diagnosis-specific effects. Here we found significant main effects for main diagnosis (*β* = −0.45; *p* < 0.001), PVS (*β* = −0.30; *p* < 0.01) and NVS (*β* = 0.31; *p* < 0.001) while SP was only significant in the robust model (*β* = 0.18; *p* = 0.07; HC robust: *p* < 0.01). Other interactions included were not found to be significant. Even though R^2^ only increased by 0.01, model m2 had a significantly better fit [*F*(4,849) = 3.231, *p* = 0.05] than model m1. To check for multicollinearity in model m2, we calculated the variance inflation factors (GVIFs) for each predictor. This involved combining the main effect of the predictor with any interactions it has with other predictors in the model. The VIFs for PVS (VIF = 136.68), NVS (VIF = 15.09), and SP (VIF = 193.77) largely exceeded the cut-off. Consequently, we analyzed domain-specific models (m3-6) with main diagnosis, separate domain covariates and their associated interaction as predictors. Table 3 includes the results of models 3–6, which indicate significant domain-by-disease severity interactions for all domains. Figure 1 shows the interaction plots of the fitted values from these separate models for the four domains.

**Table 3:** Results of models 0–6.

Model no: equation	Main diagnosis *β* (*p*)	Domain factor score *β* (*p*)	Interaction *β* (*p*)	Adj. *R*^2^	AIC	*F*-test
m0: DS ~ Dia	−0.43 ***	-	–	0.19	2515.21	*F*(1,857) = 198.7***
m1: DS ~ Dia + PVS + NVS + CS+ SP	−0.42 ***	PVS: −0.37 *** NVS: 0.30 *** CS: −0.02 (ns) SP: 0.18 ***	–	0.40	2254.34	F(5,853) = 116.8***
m2: DS ~ Dia + PVS + NVS + CS + SP + PVSxDia + NVSxDia + CSxDia + SPxDia	−0.45 ***	PVS: −0.30 ** NVS: 0.31 *** CS: −0.03 (ns) SP: 0.18 (0.07)^a^	PVSxDia: −0.09 (ns) NVSxDia: −0.09 (ns) CSxDia: 0.004 (ns) SPxDia: −0.11 (ns)	0.41	2249.36	F(9,849) = 67.0***
m3: DS ~ Dia + PVS + PVSxDia	−0.51 ***	−0.35 ***	−0.15 ***	0.39	2277.51	F(3,855) = 180.2***
m4: DS ~ Dia + NVS + NVSxDia	−0.38 ***	0.39 ***	0.09 ** ^b^	0.39	2275.22	*F*(3,855) = 181.4***
m5: DS ~ Dia + CS + CSxDia	−0.44 ***	−0.12 ***	−0.004 (ns)	0.20	2503.26	*F*(3,855) = 72.67***
m6: DS ~ Dia + SP + SPxDia	−0.48 ***	−0.32 ***	−0.16 ***	0.37	2303.22	*F*(3,855) = 166.5***

DS, disease severity; Dia, main diagnosis (AD/MDD); PVS, Positive Valence Systems; NVS, Negative Valence Systems; CS, Cognitive Systems; SP, Social Processes; *x*, by (in interaction terms); ns, not significant. *p < 0.05, **p < 0.01, ***p < 0.001; ^a^with robust HC analysis *p* < 0.05; ^b^with robust HC analysis *p* = 0.07.

We found significant main effects for diagnosis and the respective domain in all four models (m3-m6). Specifically, PVS was significantly associated with disease severity in both AD and MDD, but the effect was stronger in MDD. A similar picture emerged for NVS and SP. With regard to the CS domain, we found a significant negative association with disease severity as a main effect in m5. A higher score on the CS factor was associated with lower disease severity.

Considering the heteroskedasticity of the models, we performed additional robust model analyzes for all models (m0-m6). The results showed no changes in the reported results, except for the following two models: In m2 the main effect of SP at trend level became significant (*t* = 2.53, *p* = 0.012) and in m4 the interaction of main diagnosis and NVS changed from a significant effect to an effect at trend level (*t* = 1,81, *p* = 0.071). Furthermore, results did not differ when controlling for age differences and present comorbidities in our analyzed models. In the CS single domain model (m5) age additionally significantly predicted disease severity (*p* < 0.01).

## 4. Discussion

The main aim of our study was to examine the relationship between four core RDoC domains and disease severity among AD and MDD. As far as we know, this is the first study investigating these four transdiagnostic indicators on a domain level and their associations with disease severity in a transdiagnostic sample. Our first aim was to explore the relationship of PVS, NVS, CS, SP and disease severity across diagnostic categories. The results confirmed our hypotheses on this transdiagnostic relationship for all four domains, as we found significant main effects for PVS, NVS, CS and SP on disease severity within domain-specific models. For three domains, except CS, this main effect could also be found in the overall model as well. While NVS was positively associated with disease severity in our sample, PVS, CS and SP had a negative association with disease severity. Since we were able to show that some of these effects only occur within domain-specific analysis with similar *R*^2^ values, it stands to reason to assume that for AD and MDD, especially the effects of PVS and NVS play a superior role in this relationship to disease severity within patients with AD and MDD. However, this does not necessarily imply that anxiety predicts AD and anhedonia predicts depression; specifically, both PVS and NVS predicted diseases severity across disorders, and more so in patients suffering from MDD. Thus, we could show a transdiagnostic predictive value of both domains, which corresponds to our second main aim.

For this second aim, we investigated a disorder-specific interaction between these four domains and disease severity. Our results yielded three significant interaction effects within domain-specific models. Overall, we found a stronger association of PVS, NVS and SP with disease severity in MDD in comparison to AD, despite lower disease severity in patients with MDD compared to patients with AD. Therefore, future research should aim to replicate our findings in a longitudinal design to confirm this association.

In regards to the single RDoC domains starting with PVS, we found that low PVS manifestations, representing low hedonic affect and low habituation, were associated with high symptom burden, which is consistent with previous findings of diminished PVS processing in MDD, e.g., (19, 21), and SAD and GAD, e.g., (14, 45, 53). The finding that disease severity scores were affected by low PVS manifestations most strongly in patients with MDD is also consistent with previous research that suggests PVS-related processing as a marker for MDD, e.g., (40, 42, 54).

Our results regarding NVS are also in line with previous research in AD, e.g., (25–27), and MDD, e.g., (21, 30, 31). This previous research supports our findings of a link between high symptom burden in patients with AD and MDD with high NVS manifestations, representing high levels of anxiety and behavioral inhibition. As NVS-related processing is a common dysfunction in AD and MDD, e.g., (39, 42, 55), our results are further evidence for altered NVS functioning as a transdiagnostic marker for the spectrum of depressive and anxiety disorders.

As mentioned earlier, research on the association of a latent construct CS domain with disease severity is limited. Our results are in line with previous findings on a negative association of cognitive functioning and disease severity in MDD and AD (32, 56, 57). Our findings did not reveal a disease-specific interaction for CS, represented by executive functioning, attention, and working memory, but we did find a main effect of the disorder, indicating a decreased cognitive function in patients with MDD. The lack of a significant interaction, in the presence of known disease-specific evidence for cognitive deficits in episodic memory in patients with MDD and attentional bias in patients with AD, may be due to combining variables of several different cognitive processes into one latent variable, thereby losing crucial variance. This should be closely examined in future research.

Our findings for the SP domain are consistent with previous research that has identified dysfunction in affiliation and attachment in patients with MDD, as well as dysfunction in perception and understanding of self in patients with AD, particularly GAD (5). Additionally, there is evidence of global social functioning deficits in both AD and MDD (37, 58). Previous research on the SP domain that aligned with RDoC has primarily focused on youth or adolescent samples (11, 59). Our study extends previous research on this particular domain to adult populations by identifying disease-specific and transdiagnostic associations of social processes and symptom burden within an adult sample.

As noted, the data of this present study was provided by pooling anonymized data from all FZPE consortia. Incomplete information on comorbidities and some main diagnoses resulted in the limited availability of subgroup data sets. Specifically, the comorbidity overlap in our sample may have diluted symptom specific effects on disease severity. It should be noted, however, that despite this possible limitation, we found different associations of the domains with the diagnosis-specific symptom burden. Especially for NVS, which has been associated with anxiety, our results present differential associations despite the high comorbidity of AD with MDD in the subsample. Future studies are needed to investigate PVS, NVS, CS and SP dys−/function in specific types of AD and MDD, as well as to consider comorbidities within AD and MDD. As this is a cross-sectional study, the interpretation of our results is limited. Given that the relationship of PVS, NVS, CS and SP functioning with disease severity in AD and MDD unfolds over time, no causal conclusions can be inferred. However, we reliably showed that RDoC domains are associated with disease severity across disorders.

We would also like to point out that the majority of our disease severity ratings was based on self-report. Since the RDoC domains maybe more sensitive to self-reported disease severity future research on differential effects on self-reported versus expert-based ratings of disease severity could be additionally informative. While we used LM models as a statistical method of analysis, this approach may have limited our understanding of the domain-specific relations to the disease severity burden, because we were unable to account for random effects which could have affected the results. More sophisticated models like generalized linear mixed models (GLMM) should be considered for further investigations.

Since domain factor scores were constructed using many BSI-Items and considering the presence of well documented correlations between BSI-53 (SCL-90) and other severity measures f.e., BDI-II (60) this could be considered as another limitation impacting our results. We would like to argue, that even though there is a surplus of BSI-53 items involved in the factor structure, we still measured the latent RDoC domains and not only different types of symptom burden. Model fit of the four-factor model was significantly better in comparison to a one factor model (measuring general psychopathology) and a model with independent factors (12). Additionally, if our results were solely driven by symptom burden, we would expect that the association of NVS to disease-specific AD severity would be stronger than for MDD, which is not the case.

Given the presence of possible outliers, heteroskedasticity, and multicollinearity during our analyzes, which we addressed adequately, it is important to interpret our results within the context of these specific conditions. Especially in light of multicollinearity and the change in significance with robust testing in two of the models, the associational structure between PVS, NVS and SP has to be further investigated.

Although it is still at an early stage, there is some indication from our results that a specific RDoC-based treatment may be more effective for patients with MDD. Further investigation is needed to confirm this hypothesis. Nevertheless, there is already some evidence in this direction with an RDoC-based treatment called ENGAGE, which targets f.e., reward processing (PVS) and has shown promising results in improving outcomes for patients with MDD (61). Therefore, further development and implementation of RDoC-based disease-specific treatments could lead to more tailored and effective interventions for all mental disorders. Overall, our findings suggest that a more nuanced transnosological understanding of mental disorders’ underlying mechanisms and dimensions is needed to inform the development of more effective treatment.

In Conclusion, our key results demonstrate a strong association between symptom burden in patients with AD and MDD and latent RDoC indicators (PVS, NVS, CS, and SP) in a transdiagnostic way. There is also evidence for a disease-specific association between PVS, NVS and SP, which requires future research to further understand the association of PVS, NVS and SP with disease severity, hopefully informing specific treatment options in the future (62).

## Data availability statement

The datasets presented in this article are not readily available because they are not available publicly based on local and national data protection regulations. All original data are on record and accessible to inspection. Requests to access the datasets should be directed to michael.rapp@uni-potsdam.de.

## Ethics statement

The studies involving human participants were reviewed and approved by Potsdam Research Ethics Committee, University of Potsdam. The patients/participants provided their written informed consent to participate in this study.

## Author contributions

BF, SB, and MT managed the literature searches and analyzes. BF, MT, SB, and MR undertook the statistical analysis. BF wrote the first draft of the manuscript. All authors designed the study, wrote the protocol, and contributed to the article and approved the submitted version.

## Funding

This work was supported by the Phenotypic, Diagnostic and Clinical Domain Assessment Network Germany (PD-CAN) research consortium (FKZ 01EE1406I) and the following research consortia AERIAL (01EE1406), APIC (01EE1405), ASD-Net (01EE1409), ESPRIT (01EE1407), GCBS (01EE1403), ESCAlife (01EE1408), BipoLife (01EE1404), OptiMD (01EE1401), and PROTECT-AD (01EE1402) funded by the German Federal Ministry of Education and Research (BMBF; http://www.Bmbf.de). Open Access-Publication funded by the Deutsche Forschungsgemeinschaft (DFG, German Research Foundation) – Project number 491466077.

## Conflict of interest

FP is a member of the European Scientific Advisory Board of Brainsway Inc., Jerusalem, Israel, and the International Scientific Advisory Board of Sooma, Helsinki, Finland. He has received speaker’s honoraria from Mag&More GmbH, the neuroCare Group, Munich, Germany, and Brainsway Inc. His lab has received support with equipment from neuroConn GmbH, Ilmenau, Germany, Mag&More GmbH and Brainsway Inc. TB served in an advisory or consultancy role for eye level, Infectopharm, Lundbeck, Medice, Neurim Pharmaceuticals, Oberberg GmbH, Roche, and Takeda. He received conference support or speaker’s fee by Janssen, Medice and Takeda. He received royalities from Hogrefe, Kohlhammer, CIP Medien, Oxford University Press; the present work is unrelated to these relationships.

The remaining authors declare that the research was conducted in the absence of any commercial or financial relationships that could be construed as a potential conflict of interest.

## Publisher’s note

All claims expressed in this article are solely those of the authors and do not necessarily represent those of their affiliated organizations, or those of the publisher, the editors and the reviewers. Any product that may be evaluated in this article, or claim that may be made by its manufacturer, is not guaranteed or endorsed by the publisher.

## Abbreviations

AD: Anxiety Disorders
MDD: (Major) Depressive Disorder
SAD: Social Anxiety Disorder
GAD: Generalized Anxiety Disorder
PD-CAN: Phenotypic, Diagnostic and Clinical Domain Assessment Network Germany
FZPE: German research network for mental disorders [Forschungszentrum zu psychischen Erkrankungen]
PVS: Positive Valence Systems
NVS: Negative Valence Systems
CS: Cognitive Systems
SP: Social Processes
