# Supplementary material for: The associations of Positive and Negative Valence Systems, Cognitive Systems and Social Processes on disease severity in anxiety and depressive disorders
Source: Front Psychiatry. 2023 Jun 16;14:1161097. doi: 10.3389/fpsyt.2023.1161097 (PMC10313476; doi:10.3389/fpsyt.2023.1161097)
Supplement: Supplementary file 1 [file Table_1.pdf]

Supplementary Material

Table S1: *Sampling and eligibility criteria of the FZPE subsample*

**Consortium: coordination center (PI, location), *N*** **Inclusion criteria**	**Exclusion criteria**
**PROTECT-AD (Providing tools for effective care and treatment of anxiety disorders): Wittchen, TU Dresden, *N*=600**	
Current primary diagnosis: agoraphobia with or without panic disorder, social phobia, multiple specific (isolated) phobias, panic disorder (CIDI); outpatient status; age: 15–70 years; HAM-A >18; CGI> 3; written informed consent; ability to attend sessions and language competence	(1) any current DSM-V psychotic or substance use disorder (except nicotine); concomitant psychological or psychiatric treatment (psychopharmacological medication was allowed, if dosing was stable (for at least 3 months) and the medication was considered appropriate by the monitoring study clinician); acute suicidality; general medical contraindications; mono-symptomatic specific phobia.
**ESCAlife (Evidence-based, stepped care of ADHD along the lifespan), ESCAlate: Banaschewski, CIMH Mannheim; *N*=1**	
ADHD (DSM-V); 16-45 y., m/f	(1) psychiatric disorders; current alcohol or drug dependence; common comorbidities (e.g., CD, PD excl. ASPD); no exclusion if AD, mild to moderate MDD, or SUD in remission; severe heart disease; epilepsy; (2) psychotropics or ADHD medication: ≥4-week wash-out period prior study participation; (3) IQ<80; insufficient language skills; pregnancy or breast-feeding
**BipoLife (Improving the detection and treatment of BD): Bauer, University Hospital, TU Dresden, *N*=27**	
Dresden: 15-35 y., m/f; Risk group I: consultation of an early detection center; the presence of ≥1 risk factors for BD [a]; Risk group II: in- or outpatients with a depressive syndrome (MDD, PDD, mDD, RBD, AjD with depressed mood, unspecified)	Dresden: (1) primary diagnosis of BD, SZ, SZA, AD, OCD, or SUD; acute suicidality; (3) limited ability to comprehend the study; implied expressed negative declaration of intent to participate in the study by a minor
**OptiMD (Novel strategies for the optimized treatment of major depression): Rupprecht, University of Regensburg, *N*=140**	
Regensburg, Munich, Heidelberg: inpatients with depressive syndrome (HAM-D-21≥14; first depressive episode, recurrent MDD, BD current depressive episode, SZA, mixed AD, and MDD); ≥18 y., m/f; Caucasian origin	Regensburg, Munich, Heidelberg: (1) organic (somatic/neurological) or substance-induced cause of depressive episode; severe organic disease; (3) pregnancy or breast-feeding
Berlin: current primary MDD (ICD-10) during hospital stay; ≥18 y., m/f	Berlin: (1) CG: current psychiatric disorder (ICD-10)
**GCBS (German center for brain stimulation for psychiatric disorders): Padberg, University Hospital, LMU Munich, *N*=40**	
Munich: MDD (DSM-V; HAM-D-21≥15); current depressive episode ≤5 y. duration; in current episode no responding to ≥1 antidepressant treatment, and ≥4 weeks SSRI-intake of adequate dose; 18-65 y., m/f	Munich: (1) any other relevant psychiatric DSM-V axis-I- and/or axis-II-disorder, or unstable medical condition; acute suicidality; high degree of therapy resistance (>4 treatment attempts in the current episode); ECT in the current episode; treatment with tDCS (no single experimental sessions), DBS, or VNS; any intracranial implants; (3) investigators, site personnel directly affiliated with this study, and their immediate families; pregnancy
Berlin: MDD (DSM-V), current depressive episode ≤5 y. duration; no psychotropics (≥4 weeks) or stable SSRI-intake (≥ 4 weeks); 20-65 y., m/f	Berlin: (1) BD, AD, PTSD, ED, PD; psychotic tendencies in a lifetime; substance abuse or dependence in the past 6 months (except nicotine, caffeine); stroke (past 2 y.), epileptic seizure or diagnosed epilepsy, dementia, Parkinson's disease, Huntington's chorea, multiple sclerosis, any other neurological disease, which leads to intracranial pressure, brain lesions or higher risk for epileptic seizures; behavioral therapy or ECT in the current episode; (2) ≥4 failed medication attempts in the current episode; other psychotropic medications; (3) MRI-contraindications

Consortium: coordination center (PI, location), *N*<br>Inclusion criteria	Exclusion criteria
**APIC (Antipsychotic-induced structural and functional brain changes): Schneider, RWTH Aachen University, JARA-BRAIN, *N*=8**	
SZ (DSM-V); 18-65 y., m/f; legally competent and capable of taking part in the study	(1) severe organic disease; (2) missing or incomplete medication history; (3) MRI-contraindications; pregnancy or breast-feeding; when placed in an institution by order of public authorities or courts; dependency or employment relationship with the sponsor or investigator; concurrent clinical trial participation
**ESPRIT (Enhancing schizophrenia prevention and recovery through innovative treatments): Meyer-Lindenberg, CIMH Mannheim, *N*=43**	
SZ, MDD, BD-I, ASD; CG: no history of psychiatric disorders; 18-65 y., m/f	(1) BD-II, SUD, PD; no exclusion if comorbidities evolved as a consequence of, or were markedly less pronounced as the primary disorder; chronic physical disease; (3) MRI-contraindications

Note. *N* = 1,912 (entry data), *N* = 1,431 (after data preparation), *N* = 859 (AD/MDD subsample, used in analyses). If applicable, exclusion criteria are listed according to three categories: (1) diagnoses, treatments, (2) medications, (3) other. **Consortia**: CIMH = Central Institute of Mental Health, Mannheim; JARA-BRAIN = Jülich Aachen Research Alliance; LMU Munich = Ludwig-Maximilians-Universität München; PI = Principal investigator; RWTH= Rheinisch-Westfälische Technische Hochschule, Aachen; TU Dresden= Technische Universität Dresden. **Disorder**: AD = Anxiety disorder; ADHD = Attention deficit hyperactivity disorder; AjD = Adjustment disorder; ASD = Autism spectrum disorder; ASPD = Antisocial personality disorder; BD (-I/-II) = Bipolar disorder (Type I/II); BPD = Borderline personality disorder; CD = Conduct disorder; ED = Eating disorder; MDD = Major depressive disorder; mDD = Minor depressive disorder; OCD = Obsessive-compulsive disorder; PD = Personality disorder; PDD = Persistent depressive disorder (dysthymia, cyclothymia); PTSD = Posttraumatic stress disorder; RBD = Recurrent brief depressive disorder; SPD = Specific phobic disorder; SUD = Substance use disorder; SZ = Schizophrenia; SZA = Schizoaffective disorder. **Instrument/Manual**: DSM-V/DSM-IV= Diagnostic and Statistical Manual of Mental Disorders, Fourth/Fifth Edition (APA, 1994, 2000, 2013); CIDI = Composite International Diagnostic Interview (Robins, 1988); CGI = Clinical Global Impression Scale (Guy, 1976); HAM-A = Hamilton Anxiety Rating Scale (Hamilton, 1969; Maier et al., 1988); HAM-D-21 = Hamilton Depression Rating Scale, Version 21 (Hamilton, 1960, 1967); ICD-10 = International Classification of Diseases, 10th Revision (WHO, 2015); QIDS-C = Quick Inventory of Depression Symptomatology - Clinician rating (Rush et al., 2003); YMRS = Young Mania Rating Scale (Young et al., 1978). **Other**: DBS = Deep brain stimulation; CG = Control group; ECT = Electroconvulsive therapy**;** IQ = Intelligence quotient; m/f = male and female; MRI = Magnetic resonance imaging; SSRI = Selective serotonin reuptake inhibitor; tDCS = Transcranial direct current stimulation; VNS = Vagus nerve stimulation; y. = year(s).

[a] Listed risk factors for BD: family history, affective symptomatology/depressive syndrome, hypomanic/mood swings, disturbances of circadian rhythm/sleep, or other

The associations of Positive and Negative Valence Systems, Cognitive Systems and Social Processes on disease severity in anxiety and depressive disorders

Bernd R. Förstner*, Sarah Jane Böttger, Alexander Moldavski, Malek Bajbouj, Andrea Pfennig, André Manook, Marcus Ising, Andre Pittig, Ingmar Heinig, Andreas Heinz, Klaus Mathiak, Thomas G. Schulze, Frank Schneider, Inge Kamp-Becker, Andreas Meyer-Lindenberg, Frank Padberg, Tobias Banaschewski, Michael Bauer, Rainer Rupprecht, Hans-Ulrich Wittchen, Michael A. Rapp and Mira Tschorn

*Corresponding author: Bernd R. Förstner: bernd.forstner@uni-potsdam.de
