# Supplementary material for: The associations of Positive and Negative Valence Systems, Cognitive Systems and Social Processes on disease severity in anxiety and depressive disorders
Source: Front Psychiatry. 2023 Jun 16;14:1161097. doi: 10.3389/fpsyt.2023.1161097 (PMC10313476; doi:10.3389/fpsyt.2023.1161097)
Supplement: Supplementary file 2 [file Table_2.pdf]

Supplementary Material

Table S2: *Diagnostic information of the subsample patient groups*

**Diagnosis**	**ICD-10 code**	***N***	***DS instrument***
**Major depressive disorder (MDD)**		**257**	
MDD, single episode	F32	8	BDI-II, HAM-D21
MDD, single episode, mild	F32.0	1	HDRS-21
MDD, single episode, moderate	F32.1	27	BDI-II, CGI, HAM-D21
MDD, single episode, severe without psychotic symptoms	F32.2	42	BDI-II, CGI, HAM-D21, HDRS-D21, IDS-C30
MDD, single episode, severe with psychotic symptoms	F32.3	3	BDI-II, HAM-D21, MADRS
Other depressive episodes	F32.8	1	BDI-II
MDD, single episode, unspecified	F32.9	1	HAM-D21
MDD, single episode or recurrent, unspecified	F32/3 3	1	HAM-D21
MDD, recurrent, current episode unspecified	F33	20	HAM-D21, IDS-C30, MADRS
MDD, recurrent, current episode mild	F33.0	6	HAM-D21, HDRS-D21,IDS-C30
MDD, recurrent, current episode moderate	F33.1	40	BDI-II, CGI, HAM-D21, HDRS-D21, IDS-C30
MDD, recurrent, current episode severe without psychotic symptoms	F33.2	98	BDI-II, CGI, HAM-D21, HDRS-D21
MDD, recurrent, current episode severe with psychotic symptoms	F33.3	6	BDI-II, CGI
MDD, recurrent, currently in remission	F33.4	2	HAM-D21
PDD, dysthymia	F34.1	1	HAM-D21
**Anxiety disorders (AD)**		**602**	HAM-A, GAF
AD, Agoraphobia without panic disorder	F40.0	30	HAM-A
AD, Agoraphobia with panic disorder	F40.01	280	HAM-A
AD, Social phobia	F40.1	182	HAM-A, GAF
AD, multiple Specific (isolated) phobias	F40.2	48	HAM-A
Panic disorder	F41.0	62	HAM-A, GAF

Note. AD = Anxiety disorder; MDD = Major depressive disorder; PDD = Persistent depressive disorder. Instrument: BDI-II = Beck Depression Inventory-II (Beck and Steer, 1987); CGI-S = Clinical Global Impressions Scale - Severity of Illness (Guy, 1976); GAF = Global Assessment of Functioning Scale (Aas, 2010; APA, 1994; Rey et al., 1995); HAM-A = Hamilton Anxiety Scale (Hamilton, 1969; Maier et al., 1988); HAM-D-21 = Hamilton Rating Scale for Depression (Hamilton, 1960, 1967); IDS-C-30 = 30-Item Inventory of Depressive Symptomatology - Clinician Rating (Rush et al., 1996); MADRS = Montgomery–Åsberg Depression Rating Scale (Montgomery and Asberg, 1979).

The associations of Positive and Negative Valence Systems, Cognitive Systems and Social Processes on disease severity in anxiety and depressive disorders

Bernd R. Förstner*, Sarah Jane Böttger, Alexander Moldavski, Malek Bajbouj, Andrea Pfennig, André Manook, Marcus Ising, Andre Pittig, Ingmar Heinig, Andreas Heinz, Klaus Mathiak, Thomas G. Schulze, Frank Schneider, Inge Kamp-Becker, Andreas Meyer-Lindenberg, Frank Padberg, Tobias Banaschewski, Michael Bauer, Rainer Rupprecht, Hans-Ulrich Wittchen, Michael A. Rapp and Mira Tschorn

*Corresponding author: Bernd R. Förstner: bernd.forstner@uni-potsdam.de
