# Supplementary material for: The associations of Positive and Negative Valence Systems, Cognitive Systems and Social Processes on disease severity in anxiety and depressive disorders
Source: Front Psychiatry. 2023 Jun 16;14:1161097. doi: 10.3389/fpsyt.2023.1161097 (PMC10313476; doi:10.3389/fpsyt.2023.1161097)
Supplement: Supplementary file 3 [file Table_3.pdf]

Supplementary Material

Table S3: *Construct-level indicators and factor loadings of the domain-level PVS, NVS, CS and SP factors*

**RDoC factor,** construct	**Indicator**	**Self-report/ behave. Assess.**	**Item(s)**	***β***
**PVS**				
Reward responsiveness	Anhedonia	BSI-53 item [d]	18	-.76
Reward responsiveness	Hedonic affect	PANAS items [c]	1, 3, 4, 17	.55
Reward learning	Habituation	BSI-53 items [b]	15r, 26r, 27r	.81
**NVS**				
Potential threat	Anxiety	BSI-53 subscale	1, 12, 19, 38, 45, 49	.91
Potential threat	Phobic anxiety	BSI-53 subscale	8, 28, 31, 43, 47	.81
Potential threat	Somatization	BSI-53 subscale	2, 7, 23, 29, 30, 33, 37	.75
Potential threat	Anxiety-based BIS	BIS/BAS items [a]	2r, 8, 22r, 24	.49
**CS**				
Attention	Raw score Trail Making Test – Version A	TMT A	Time raw score	-.80
Cognitive control	Trail Making Test – Version B	TMT B	Time raw score	-.79
Working memory	Digit Symbol Substitution Test	DSST	Raw test score	.69
**SP**				
Perception and understanding of others	Hostility	BSI-53 subscale	6, 13, 40, 41, 46	-.76
Affiliation and attachment	Social Anhedonia	BSI-53	14	-.76
Affiliation and attachment	Interpersonal sensitivity	BSI-53 subscale	20, 21, 22, 42	-.91
Affiliation and attachment	Friendships	WHO-DAS-20	11r	.53
Perception and understanding of self	Paranoid ideation	BSI-53 subscale	4, 10, 24, 48, 51	-.79

Note. Indicators and factor loadings of PVS, NVS, CS and SP factors, which resulted from a CFA on the latent RDoC structure using behavioral and self-report assessments in a transdiagnostic sample. General CFA model fit was evaluated using the following fit indices: CFI = .93, TLI = .92, RMSEA = .077. All factor loadings were highly significant ($p < .001$). **RDoC**: PVS = Positive valence systems; NVS = Negative valence systems; CS = Cognitive systems; SP = Systems of social processes; RDoC = Research Domain Criteria. **CFA**: *β* = Factor loadings; CFI = Comparative Fit Index; r= Reversed items; RMSEA = Root Mean Square Error of Approximation; TLI = Tucker-Lewis Index. **Instrument**: BIS/BAS = Behavioral Inhibition System, Behavior Activation System Scales (Strobel et al., 2001); BSI-53 = Brief Symptom Inventory-53 (Franke, 2000); PANAS = Positive and Negative Affect Schedule (Breyer & Bluemke, 2016); WHO-DAS-20 = ; TMT A/B = Trail making Test A/B; DSST = Digit Symbol substitution Test.

[a] Selected items from the BIS subscale forming a mean score of anxiety-based BIS

[b] Selected items from the BSI-53 obsessive-compulsive subscale forming a mean score of habituation

[c] Selected items from the PANAS positive affect subscale forming a mean score of hedonic affect

[d] Selected single item from the BSI-53 using the raw value to measure anhedonia

The associations of Positive and Negative Valence Systems, Cognitive Systems and Social Processes on disease severity in anxiety and depressive disorders

Bernd R. Förstner*, Sarah Jane Böttger, Alexander Moldavski, Malek Bajbouj, Andrea Pfennig, André Manook, Marcus Ising, Andre Pittig, Ingmar Heinig, Andreas Heinz, Klaus Mathiak, Thomas G. Schulze, Frank Schneider, Inge Kamp-Becker, Andreas Meyer-Lindenberg, Frank Padberg, Tobias Banaschewski, Michael Bauer, Rainer Rupprecht, Hans-Ulrich Wittchen, Michael A. Rapp and Mira Tschorn

*Corresponding author: Bernd R. Förstner: bernd.forstner@uni-potsdam.de
