# Supplementary material for: The associations of Positive and Negative Valence Systems, Cognitive Systems and Social Processes on disease severity in anxiety and depressive disorders
Source: Front Psychiatry. 2023 Jun 16;14:1161097. doi: 10.3389/fpsyt.2023.1161097 (PMC10313476; doi:10.3389/fpsyt.2023.1161097)
Supplement: Supplementary file 4 [file Table_4.pdf]

Supplementary Material

Table S4: *Normative z-transformation of disease severity scores*

Test	Range [a]	Group	Reference population			Source	No. used in sample (N=859)
			Sample	*N*	*M* ± *SD*		n, (%)
**Disease-specific self-report scales**							**AD: 0 (0%); MDD: 40 (100%)** [b]
BDI-II	0-63	MDD	MDD and PDD outpatients	4,019	30.8 ± 10.52	Schulte-van Maaren et al., 2013	40 (4.7)
**Disease-specific observer ratings**							**AD: 600 (81.7); MDD: 134 (18.3)** [b]
MADRS	0-60	MDD	MDD and PDD outpatients	4,627	23.44 ± 7.75	Schulte-van Maaren et al., 2013	6 (0.7)
HAM-A	0-56	AD	GAD outpatients	688	24.6 ± 7.0	Allgulander et al., 2007	600 (69.8)
HAM-D-21	0-66	MDD	MDD and BD inpatients	768	25.9 ± 6.80	Hennings et al., 2009	117 (13.6)
IDS-C-30	0-84	MDD	MDD outpatients	544	37.7 ± 13.80	Rush et al., 2006	11 (1.3)
**Expert-based global rating scales**							**AD: 2 (2.3); MDD: 83 (97.7)** [b]
CGI-S	1-7	MDD	MDD patients	6,895	4.5 ± 0.8	Leucht et al., 2013	82 (9.6)
GAF	1-100	AD	SAD outpatients	644	49.9 ± 9.3	Kelly et al., 2013	2 (0.2)
GAF	1-100	MDD	MDD and PDD outpatients	1,489	57.3 ± 6.8	van Noorden et al., 2012	1 (0.1)

Note. The individual disease severity scores were converted into z-values by means and standard deviations of normative data from adult clinical samples. *M* = Mean; *SD* = Standard deviation. **Disorder**: AD = Anxiety disorder; BD = Bipolar disorder; GAD = General anxiety disorder; MDD = Major depressive disorder; PDD = Persistent depressive disorder (dysthymia). **Instrument**: BDI-II = Beck Depression Inventory-II (Beck and Steer, 1987); CGI-S = Clinical Global Impressions Scale - Severity of Illness (Guy, 1976); GAF = Global Assessment of Functioning Scale (Aas, 2010; APA, 1994; Rey et al., 1995); HAM-A = Hamilton Anxiety Scale (Hamilton, 1969; Maier et al., 1988); HAM-D-21 = Hamilton Rating Scale for Depression (Hamilton, 1960, 1967); IDS-C-30 = 30-Item Inventory of Depressive Symptomatology - Clinician Rating (Rush et al., 1996); MADRS = Montgomery–Åsberg Depression Rating Scale (Montgomery and Asberg, 1979).

[a] Higher test scores indicate higher levels of disease severity, except for GAF scores ranging from 100 (extremely high functioning) to 1 (severely impaired), these scores were reversed.

[b] Percentage of the respective Ratings

The associations of Positive and Negative Valence Systems, Cognitive Systems and Social Processes on disease severity in anxiety and depressive disorders

Bernd R. Förstner*, Sarah Jane Böttger, Alexander Moldavski, Malek Bajbouj, Andrea Pfennig, André Manook, Marcus Ising, Andre Pittig, Ingmar Heinig, Andreas Heinz, Klaus Mathiak, Thomas G. Schulze, Frank Schneider, Inge Kamp-Becker, Andreas Meyer-Lindenberg, Frank Padberg, Tobias Banaschewski, Michael Bauer, Rainer Rupprecht, Hans-Ulrich Wittchen, Michael A. Rapp and Mira Tschorn

*Corresponding author: Bernd R. Förstner: bernd.forstner@uni-potsdam.de
